# A comparison of two registry-based systems for the surveillance of persons hospitalised with COVID-19 in Norway, February 2020 to May 2022

**DOI:** 10.2807/1560-7917.ES.2023.28.33.2200888

**Published:** 2023-08-17

**Authors:** Robert Whittaker, Salla Toikkanen, Katharine Dean, Trude Marie Lyngstad, Eirik Alnes Buanes, Hilde Kløvstad, Trine Hessevik Paulsen, Elina Seppälä

**Affiliations:** 1 Department of Infection Control and Vaccines, Norwegian Institute of Public Health, Oslo, Norway; 2 Department of Infection Control and Preparedness, Norwegian Institute of Public Health, Oslo, Norway; 3 Department of Anaesthesia and Intensive Care, Haukeland University Hospital, Bergen, Norway; 4 Norwegian Intensive Care and Pandemic Registry, Haukeland University Hosspital, Bergen, Norway

**Keywords:** COVID-19, surveillance, hospitalisation, Norway, electronic health registry, International Classification of Diseases

## Abstract

**Background:**

The surveillance of persons hospitalised with COVID-19 has been essential to ensure timely and appropriate public health response. Ideally, surveillance systems should distinguish persons hospitalised with COVID-19 from those hospitalised due to COVID-19.

**Aim:**

We compared data in two national electronic health registries in Norway to critically appraise and inform the further development of the surveillance of persons hospitalised with COVID-19.

**Method:**

We included hospitalised COVID-19 patients registered in the Norwegian Patient Registry (NPR) or the Norwegian Pandemic Registry (NoPaR) with admission dates between 17 February 2020 and 1 May 2022. We linked patients, identified overlapping hospitalisation periods and described the overlap between the registries. We described the prevalence of International Classification of Diseases (ICD-10) diagnosis codes and their combinations by main cause of admission (clinically assessed as COVID-19 or other), age and time.

**Results:**

In the study period, 19,486 admissions with laboratory-confirmed COVID-19 were registered in NoPaR and 21,035 with the corresponding ICD-10 code U07.1 in NPR. Up to late 2021, there was a 90–100% overlap between the registries, which thereafter decreased to < 75%. The prevalence of ICD-10 codes varied by reported main cause, age and time.

**Conclusion:**

Changes in patient cohorts, virus characteristics and the management of COVID-19 patients from late 2021 impacted the registration of patients and coding practices in the registries. Using ICD-10 codes for the surveillance of persons hospitalised due to COVID-19 requires age- and time-specific definitions and ongoing validation to consider temporal changes in patient cohorts and virus characteristics.

**Key public health message**

**What did you want to address in this study?**

During the COVID-19 pandemic, surveillance systems that collect data on people hospitalised with COVID-19 have been essential to ensure timely and appropriate public health response. To critically appraise and further develop the surveillance of people hospitalised with COVID-19, we compared data on hospitalised COVID-19 patients from two national health registries in Norway from February 2020 to May 2022.

**What have we learnt from this study?**

Both registries recorded a high proportion of COVID-19 patients. However, this proportion declined from late 2021, coinciding with high vaccination coverage, emergence of the milder Omicron virus variant and changes in the management of hospitalised COVID-19 patients. The overlap between hospital diagnosis codes and the clinician’s assessment of whether a patient was hospitalised due to COVID-19 varied by age and time.

**What are the implications of your findings for public health?**

National health registry data provided an accurate picture of people hospitalised with COVID-19 in Norway. However, there are challenges with using health registries for this surveillance. This comparison has improved our understanding of the data in each registry through different phases of the pandemic and can inform the ongoing development of surveillance systems for COVID-19 and in preparation for future pandemics.

## Introduction

Coronavirus disease 2019 (COVID-19) is caused by infection with severe acute respiratory syndrome coronavirus 2 (SARS-CoV-2), and the spectrum of disease may range from asymptomatic infection to severe respiratory failure. Since the start of the COVID-19 pandemic in early 2020, the surveillance of persons hospitalised with COVID-19 has been essential to ensure timely and appropriate public health response. Data from this surveillance have informed understanding of the trend and severity of the pandemic and also been used to study factors associated with severe disease [1-7]. Different approaches to this surveillance have emerged around Europe. Examples include data collection integrated with infectious disease notifications [2,8], systems based on pre-existing national patient registries [9-11], systems for the surveillance of severe acute respiratory infection (SARI) [8,12] and implementation of a voluntary patient-level clinical survey [13].

During the first 2 years of the COVID-19 pandemic, concern about healthcare capacity led to strict infection prevention and control measures worldwide. However, with high vaccine effectiveness against severe disease found to be more sustained than against infection [3,4] and the global spread of the less virulent Omicron variant (Phylogenetic Assignment of Named Global Outbreak (Pango) lineage designation B.1.1.529) from late 2021 [5-7], many countries scaled back non-pharmaceutical interventions and testing for SARS-CoV-2. These developments increased community transmission and reduced the proportion of COVID-19 cases diagnosed, but also reduced the proportion of cases who developed severe disease. This increased the importance of the surveillance of hospitalisation, not only with, but due to COVID-19, as a positive test for SARS-CoV-2 could be incidental in a larger proportion of hospitalised persons. Some countries have developed indicators for this surveillance [9,14].

Norway (population 5.4 million) confirmed its first case of COVID-19 in February 2020 and has experienced several waves of SARS-CoV-2 infection, including those driven by the Alpha (Pango lineage designation B.1.1.7), Delta (Pango lineage designation B.1.617.2) and Omicron variants [15]. Throughout the pandemic, Norway has been in the unique position of having two national electronic health registries (EHR) for the surveillance of persons hospitalised with COVID-19, one of which also disaggregates admissions due to COVID-19 based on a clinical assessment. To critically appraise and inform the further development of the surveillance of persons hospitalised with COVID-19, we linked and compared data in the two EHR from the first 2 years of the pandemic.

## Methods

### Data sources

#### Norwegian Pandemic Registry (NoPaR)

The Norwegian Pandemic Registry (NoPaR) has been the primary data source in Norway for the surveillance of persons hospitalised with COVID-19. It is a national clinical registry established in March 2020 as an expansion of the Norwegian Intensive Care Registry [16]. Data on hospital stays for patients who test positive for SARS-CoV-2 by PCR are collected in NoPaR. Patients admitted with sequelae of COVID-19 are registered if they tested positive < 3 months before admission. Patients readmitted for non-COVID-19-related causes are not registered if they are not isolated. Outpatient visits are not registered [17]. All Norwegian hospitals report to NoPaR. Reporting is mandatory and reporting criteria in the study period were consistent. Data collected include demographic characteristics (age, sex, underlying comorbidities), time of admission and discharge, and clinical condition and treatment at admission and during the stay of the patient. The reported main cause of hospitalisation (COVID-19 or other) is the physician’s clinical assessment. For patients with underlying comorbidities, COVID-19 is reported as the main cause if it contributed to a worsening of the underlying condition that necessitated hospitalisation. International Classification of Diseases (ICD) diagnosis codes [18] are not registered.

#### Norwegian Patient Registry (NPR)

The Norwegian Patient Registry (NPR) is a central health registry established in 1997 and contains data on all patients who are referred to or receive specialist healthcare at a hospital, outpatient clinic or contracted specialist in Norway [19]. All Norwegian hospitals report to NPR, and reporting is mandatory [20]. At discharge, at the latest, the ICD diagnosis codes are registered and related to the reimbursement claims of the hospitals. Throughout the pandemic, national guidelines have recommended the use of the ICD-10 code U07.1 (COVID-19, virus identified) when COVID-19 is laboratory-confirmed, regardless of the clinical illness of the patient, and that the code is registered in addition and secondary to relevant codes for the clinical illness (e.g. pneumonia) [21]. Throughout the pandemic and as of March 2023, national guidelines recommend PCR to confirm all patients who seek healthcare for COVID-19 and in all cases where confirmation is important for differential diagnosis and choice of treatment.

#### The Norwegian surveillance system for communicable diseases laboratory database (MSIS-labdatabase)

The Norwegian surveillance system for communicable diseases laboratory database (MSIS-labdatabase) [22] contains results for microbiological samples analysed for SARS-CoV-2 in all medical microbiological laboratories in Norway.

#### The national population registry

The national population registry includes demographic and administrative information on all individuals who reside or have resided in Norway [23]. This includes the national identification number, which was essential in our study for analyses that required linking registries.

### Data access

We obtained data through the emergency preparedness registry for COVID-19 (Beredt C19), housed at the Norwegian Institute of Public Health. This registry contains individual-level, daily updated data from different central health registries, national clinical registries and other relevant national registries [24]. Owing to the principle of data minimisation, researchers in Beredt C19 are only given access to full ICD-10 codes from NPR that are necessary for performing the required analyses. The full ICD-10 codes we had access to are presented in Supplement 1. We extracted data on 12 May 2022.

### Data analysis

#### Patient cohorts

We included patients registered in NPR or NoPaR with hospital admission dates between 17 February 2020 and 1 May 2022. From NPR, we included overnight stays (both urgent and elective admissions). We considered individual stays for the same patient with < 2 days between discharge and subsequent admission to be part of the same hospitalisation period, regardless of the main cause (NoPaR) or diagnosis codes (NPR) registered for subsequent admissions. An individual could thus have several COVID-19 hospitalisation periods if there were ≥ 2 days between stays. We calculated the age of the patients at the start of the hospitalisation period from birth dates in the national population register and defined four age groups (0–17, 18–54, 55–74 and ≥ 75 years). We defined four time periods which are presented in Table 1.

**Table 1:** Defined time periods and public health measures taken during the COVID-19 pandemic, Norway, 17 February 2020–1 May 2022

Week/year	Dominant circulating virus variant [15]	COVID-19 vaccination programme [15]	Summary of major national public health measures implemented during periods of peak transmission^a^ [38]
9/2020–6/2021	Wild type/‘Wuhan’	Started week 52/2020	In mid-March 2020, public health measures were implemented, including the closure of preschools, schools and hospitality services and businesses with one-to-one customer contact, cancellation of cultural and sporting arrangements and closing of borders to non-residents. Cases, close contacts and travellers returning from areas with high transmission were obliged to quarantine. Hospitals were instructed to reduce normal operations and prepare for an influx of COVID-19 patients. The restrictions were gradually lifted from mid-April 2020, although some remained (e.g. quarantine for cases, close contacts and most travellers), as well as the general recommendations to stay home when sick, wash hands, limit social contact and maintain a 1 m distance.
7/2021–26/2021	Alpha	Second dose coverage reached 95% among persons ≥ 75 years and first dose coverage reached 66% among persons ≥ 18 years, few hospitalised COVID-19 patients vaccinated.	Mitigating measures in place. In addition to the general recommendations, quarantine for cases, close contacts and most travellers, obligatory testing on arrival for travellers, limitations on who may enter Norway, use of face masks in public spaces recommended or required, alcohol serving banned or restricted, many clubs and activities for adults closed, limits on number of guests allowed at private homes, limits on number of people allowed at indoor and outdoor events and in businesses with one-to-one contact. Preschools and schools remained open, but working or studying from home was recommended where possible for workplaces and tertiary educational institutions. Hospitals were under pressure but functioned within capacity with consistent criteria for admission of COVID-19 patients.
27/2021–51/2021	Delta	Second dose coverage reached 89% among persons ≥ 18 years, increasing proportion of hospitalised COVID-19 patients vaccinated with at least two doses.	Measures similar to the Alpha period aligned with disease burden and vaccination coverage, but with use of COVID-19 passes to relax restrictions for those with documented vaccination or previous infection, such as exemption from quarantine for close contacts and arriving travellers.
52/2021–17/2022	Omicron	Third dose coverage reached 90% among persons ≥ 75 years and 66% among persons ≥ 18 years, majority of hospitalised COVID-19 patients vaccinated with three doses.	Initially similar to the Alpha and Delta periods, but moving from a control strategy to a preparedness strategy with the gradual relaxation of measures and return to a ‘normal every-day’. For example, all statutory measures, including requirements for face masks, a 1 m distance and the obligation of cases to quarantine were removed on 12 February 2022.

^a^ The range and timing of the measures implemented were not always uniform across Norway, particularly during periods when the level of transmission differed notably between regions. A comprehensive timeline is presented in [38].

#### System coverage

We identified overlapping hospitalisation periods in NPR and NoPaR. To explore what proportion of all patients with a recent positive SARS-CoV-2 test were registered in NoPaR or with the ICD-10 code U07.1 in NPR, we also linked NPR with positive SARS-CoV-2 PCR tests in MSIS-labdatabase taken ≤ 14 days before admission until discharge. We chose 14 days to ensure we identified all patients with recent positive tests that could reasonably be expected to be registered with U07.1 or in NoPaR, while 14 days was also the cut-off used in comparable registry-based surveillance systems in other countries [9,14] and studies on variant severity [6]. To identify admissions with COVID-19 in NPR, we used three definitions: (i) positive PCR test, (ii) U07.1 and (iii) positive PCR test and U07.1. For NoPaR, we included all admissions. We calculated the proportion of admissions with COVID-19 in NPR that overlapped with admissions in NoPaR and the proportion of admissions in NoPaR that overlapped with U07.1 admissions in NPR. Proportions are presented as 4-week moving averages. Among U07.1 patients, we also described the proportion with a positive PCR test > 14 days before admission or positive rapid antigen test ≤ 14 days before admission until discharge.

#### Hospitalisation due to COVID-19

To study the association between ICD-10 diagnosis codes and the clinical assessment of main cause of admission, we retrieved ICD-10 codes from NPR for the first overlapping hospitalisation period for each patient in NoPaR. We only included the first overlapping period, as the similarity of multiple hospitalisations for a particular patient could distort the distribution. The ICD-10 codes available included full codes on acute upper and lower respiratory infections (ARI), while for other codes only the first letter was available (Supplement 1). For J codes (diseases of the respiratory system), we grouped codes for pneumonia (J12–J18), other acute lower respiratory infections (J20–J22 and J80) and acute upper respiratory infections (URI) (J00–J06). We grouped the respiratory syncytial virus-specific codes for pneumonia (J12.1) and acute lower respiratory infections (J20.5 and J21.0) separately, as respiratory syncytial virus should be distinguished in an integrated (meaning covering several diseases) surveillance system for viral respiratory infections [25]. Other respiratory diseases (J codes, excluding J00–J22 and J80) were grouped according to the first letter of the diagnosis code (J (non-ARI)). We calculated the prevalence of all different ICD-10 codes and their combinations by reported main cause of admission (COVID-19 or other), age group and period. For efficiency, we used an a priori algorithm (R package arules [26]). For each age group or period, we also calculated the sensitivity and specificity of selected diagnosis code combinations for representing U07.1 patients’ main cause of admission. We also present the trend in new admissions for patients with COVID-19 as main cause in NoPaR and selected ICD-10 code combinations in NPR using data aggregated separately from each registry.

All analyses were conducted with R (version 4.0.2) [27].

## Results

### Patient cohorts

**Figure 1:**
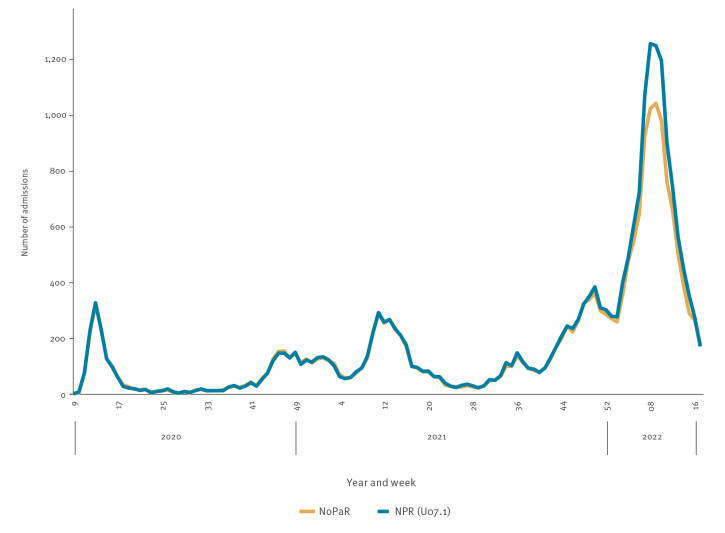
Weekly number of hospital admissions of patients with confirmed COVID-19 in the Norwegian Pandemic Registry (n = 19,486) and the Norwegian Patient Registry (n = 21,035), Norway, 17 February 2020–1 May 2022

NoPaR: Norwegian Pandemic Registry; NPR: Norwegian Patient Registry; U07.1: COVID-19, virus identified.

Between 17 February 2020 and 1 May 2022, 19,486 admissions were registered in NoPaR and 1,790,062 overnight admissions in NPR (21,035 with U07.1). The number of weekly admissions followed a similar trend in both registries. Since the beginning of 2022, NoPaR registered fewer admissions compared with U07.1 in NPR (Figure 1).

The data behind the figure are available in Supplement 2.

### System coverage

**Figure 2:**
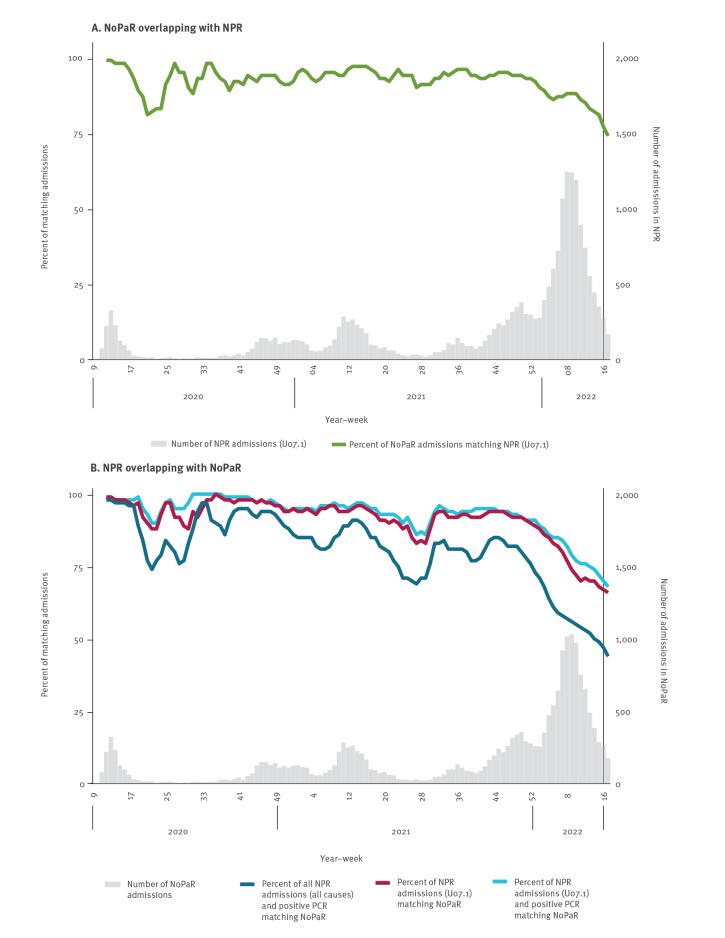
Number of COVID-19 admissions in Norwegian Pandemic Registry and Norwegian Patient Registry and a 4-week moving average of the proportion of overlapping admissions between the registries, Norway, 17 February 2020–1 May 2022

NoPaR: Norwegian Pandemic Registry; NPR: Norwegian Patient Registry; SARS-CoV-2: severe acute respiratory syndrome coronavirus 2; U07.1: COVID-19, virus identified.

Of the 19,486 admissions in NoPaR and 21,035 admissions with U07.1 in NPR, respectively, 19,250 (99%) and 20,815 (99%) had a national identification number and could be linked. Characteristics of these patients are appended in Supplement 1. Of the 19,250 admissions in NoPaR, 17,292 (90%) overlapped with a U07.1 admission in NPR, while 1,696 (8.8%) had ICD-10 codes other than U07.1 as detailed in Supplement 1. The remaining 262 (1.4%) did not overlap with an admission in NPR. The median length of stay for these 262 individuals was 3 days (interquartile range: 1–7), thus some may not have qualified as an overnight admission in NPR. Of the 20,815 U07.1 admissions in NPR, 17,307 (83%) overlapped with an admission in NoPaR. Generally, 90–100% of the hospitalisation periods overlapped between the two registries until late 2021, with exceptions predominantly in weeks with few admissions (Figure 2). From late 2021, the overlap gradually decreased to < 75%.

Panel A: 4-week moving average of the proportion of weekly admissions with confirmed COVID-19 in NoPaR that overlapped with admissions with confirmed COVID-19 (U07.1) in NPR and weekly number of admissions in NPR.

Panel B: A 4-week moving average of the proportion of weekly admissions in NPR with U07.1 and/or positive PCR test for SARS-CoV-2 ≤ 14 days before admission until discharge in the Norwegian surveillance system for communicable diseases laboratory database that overlapped with admissions with confirmed COVID-19 in NoPaR and weekly number of admissions in NoPaR.

The data behind the figure are available in Supplement 2.

Of the 20,815 U07.1 admissions in NPR, 18,918 (91%) linked to a recent positive PCR test. Of the 1,897 remaining patients (1,574 admitted from week 52/2021 (83%)), 248 (13%) had a positive PCR 15–28 days before admission, 29 (1.5%) a positive PCR 29–60 days before admission and 72 (3.8%) had a positive rapid antigen test ≤ 14 days before admission until discharge.

Of the 26,506 admissions in NPR with a positive PCR ≤ 14 days before admission until discharge, 18,918 (71%) had U07.1 registered. The proportion with U07.1 decreased from late 2021 (9,276/10,722 (87%) up to 51/2021, 9,642/15,784 (61%) from 52/2021). The proportion registered in NoPaR followed a similar pattern (9,128/10,722 (85%) up to 51/2021, 8,631/15,784 (55%) from 52/2021) (Figure 2).

### Hospitalisation due to COVID-19

**Figure 3:**
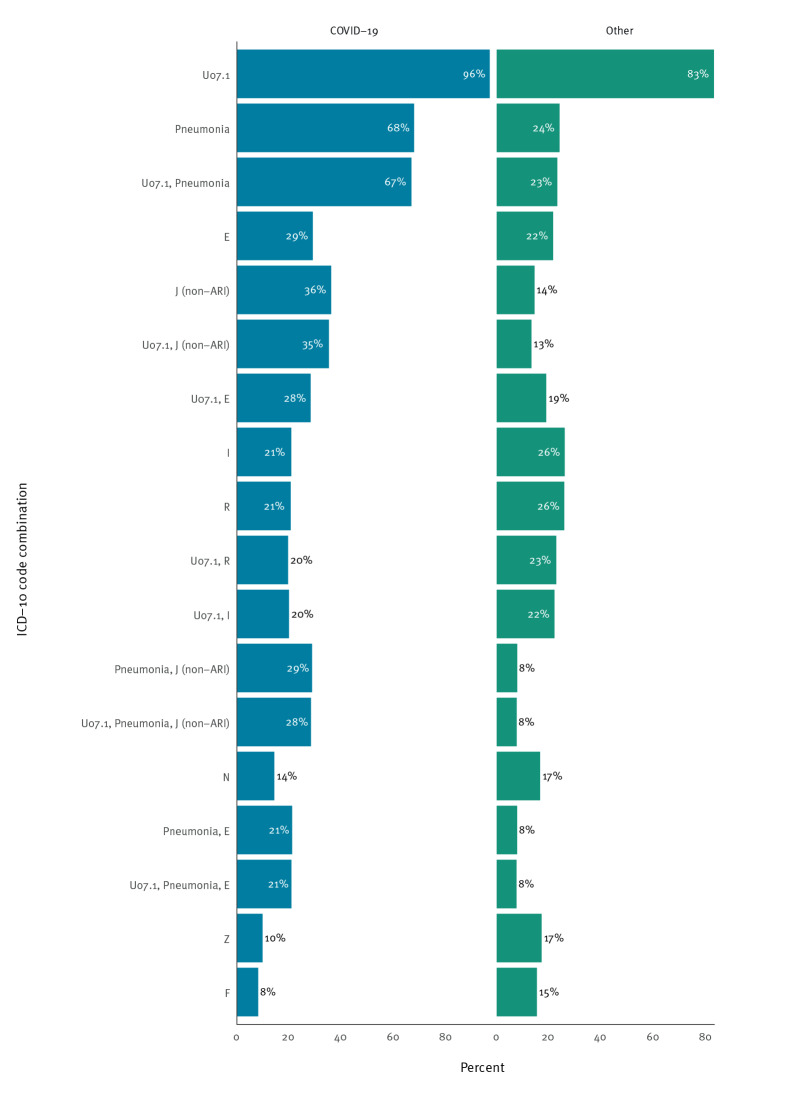
Prevalence of international classification of diseases diagnosis codes and code combinations by clinically assessed main cause of admission, Norway, 17 February 2020–1 May 2022

ARI: acute respiratory infections; ICD-10: International Classification of Diseases 10th revision.

We included 18,009 overlapping first admissions in NoPaR and NPR, excluding 163 for which the reported main cause was unknown. The prevalence of different ICD-10 code combinations by main cause are shown in Figure 3. For both admissions with main cause COVID-19 (n = 11,803) and other (n = 6,206), U07.1 was the most common code registered. For admissions with COVID-19 as main cause, 7,976 (68%) were registered with a pneumonia code and 4,244 (36%) with a J (non-ARI) code. There was more variation in the ICD-10 codes for admissions with another main cause than COVID-19. Detailed data by age group and period are available in Supplement 1. Regardless of period or main cause, there was generally a greater prominence of a wider range of codes among older than younger age groups. For patients ≥ 75 years, pneumonia followed by J (non-ARI) codes were most common among those admitted with COVID-19 as main cause across all periods, although less prominent from week 52/2021. A similar pattern was observed among patients 18–54 and 55–74 years, with increased prominence of R (symptoms, signs and abnormal clinical and laboratory findings not elsewhere classified) and Z codes (factors influencing health status and contact with health services) in later periods. Among patients 0–17 years, R codes were prominent in all time periods regardless of main cause, while the proportion of URI codes increased over time, particularly among those admitted with COVID-19 as main cause.

ICD-10 codes: E: endocrine, nutritional and metabolic diseases; F: mental, behavioural and neurodevelopmental disorders; I: diseases of the circulatory system; J: diseases of the respiratory system; N: diseases of the genitourinary system; R: symptoms, signs and abnormal clinical and laboratory findings not elsewhere classified; U07.1: COVID-19, virus identified; Z: factors influencing health status and contact with health services.

Pneumonia: ICD-10 codes J12–J18, excluding J12.1. J (non-ARI): ICD-10 codes from group J excluding J00–J22 and J80.

Only codes and code combinations with at least 15% of patients for either main cause are presented. Data on ICD-10 diagnosis codes come from the Norwegian Patient Registry and data on the main cause of admission (COVID-19 or other) from the Norwegian Pandemic Registry. Code combinations may overlap, for example patients registered with U07.1 and a pneumonia code are included in U07.1, pneumonia, and U07.1 + pneumonia. The data behind the figure are available in Supplement 2.

The sensitivity and specificity of selected ICD-10 code combinations for representing patients’ main cause of admission are presented in Table 2. For this analysis, among 18,009 first overlapping hospitalisation periods in NoPaR, we only included those with U07.1 registered (n = 16,523, 92%), to explore code combinations beyond U07.1. We explored combinations of pneumonia, J (non-ARI), URI and R codes based on those most prominent among patients admitted with COVID-19 as main cause in different age groups and periods. The prevalence of ICD-10 diagnosis codes and code combinations by main cause of admission, age group and period are provided in Supplement 1. The three code combinations including pneumonia had a higher sensitivity among patients ≥ 18 years than 0–17 years. Conversely, the combination URI or R had the lowest sensitivity among all age groups and periods, except patients 0–17 years. In the period from 52/2021 the sensitivity of all code combinations including pneumonia was lower and the specificity higher, compared with earlier periods.

**Table 2:** Sensitivity and specificity of selected international classification of diseases diagnosis code combinations for representing the main cause of admission of COVID-19 patients^a^, Norway, 17 February 2020–1 May 2022

Norwegian Patient Registry	Norwegian Pandemic Registry			Sensitivity	Specificity
Diseases and ICD-10 disease code combinations of COVID-19 patients	Diagnosis	COVID-19 as main cause	Other main causes		
		n	n	%	%
Overall					
Pneumonia	Yes	7,857	1,436	69	72
	No	3,523	3,707		
Pneumonia or J (non-ARI)	Yes	8,665	1,785	76	65
	No	2,715	3,358		
URI or R	Yes	2,829	1,545	25	70
	No	8,551	3,598		
Pneumonia or J (non-ARI) or URI or R	Yes	10,149	2,910	89	43
	No	1,231	2,233		
By age group (years)					
Age group 0–17 years					
Pneumonia	Yes	38	17	8	94
	No	413	269		
Pneumonia or J (non-ARI)	Yes	63	27	14	91
	No	388	259		
URI or R	Yes	267	125	59	56
	No	184	161		
Pneumonia or J (non-ARI) or URI or R	Yes	320	145	71	49
	No	131	141		
18–54 years					
Pneumonia	Yes	2,687	329	71	82
	No	1,085	1,536		
Pneumonia or J (non-ARI)	Yes	2,861	398	76	79
	No	911	1,467		
URI or R	Yes	921	502	24	73
	No	2,851	1,363		
Pneumonia, J (non-ARI), URI or R	Yes	3,404	822	90	56
	No	368	1,043		
55–74 years					
Pneumonia	Yes	2,847	421	76	68
	No	880	908		
Pneumonia or J (non-ARI)	Yes	3,079	533	83	60
	No	648	796		
URI or R	Yes	821	418	22	69
	No	2,906	911		
Pneumonia or J (non-ARI) or URI or R	Yes	3,411	817	92	39
	No	316	512		
≥ 75 years					
Pneumonia	Yes	2,285	669	67	60
	No	1,145	994		
Pneumonia or J (non-ARI)	Yes	2,662	827	78	50
	No	768	836		
URI or R	Yes	820	500	24	70
	No	2,610	1,163		
Pneumonia or J (non-ARI) or URI or R	Yes	3,014	1,126	88	32
	No	416	537		
By time (week/year)					
9/2020–6/2021^b^					
Pneumonia	Yes	1,917	271	77	54
	No	564	316		
Pneumonia or J (non-ARI)	Yes	2,035	303	82	48
	No	446	284		
URI or R	Yes	506	179	20	70
	No	1,975	408		
Pneumonia or J (non-ARI) or URI or R	Yes	2,275	405	92	31
	No	206	182		
7/2021–26/2021^c^					
Pneumonia	Yes	1,710	144	88	58
	No	230	195		
Pneumonia or J (non-ARI)	Yes	1,746	163	90	52
	No	194	176		
Upper respiratory infections or R	Yes	342	87	18	74
	No	1,598	252		
Pneumonia or J (non-ARI) or URI or R	Yes	1,876	222	97	35
	No	64	117		
27/2021–51/2021^d^					
Pneumonia	Yes	1,881	364	80	58
	No	476	500		
Pneumonia or J (non-ARI)	Yes	1,979	416	84	52
	No	378	448		
URI or R	Yes	494	239	21	72
	No	1,863	625		
Pneumonia or J (non-ARI) or URI or R	Yes	2,176	553	92	36
	No	181	311		
52/2021–17/2022^e^					
Pneumonia	Yes	2,349	657	51	80
	No	2,253	2,696		
Pneumonia or J (non-ARI)	Yes	2,905	903	63	73
	No	1,697	2,450		
URI or R	Yes	1,487	1,040	32	69
	No	3,115	2,313		
Pneumonia or J (non-ARI) or URI or R	Yes	3,822	1,730	83	48
	No	780	1,623		

ARI: acute respiratory infections; ICD-10: International Classification of Diseases 10th revision. URI: upper respiratory infections.

ICD-10 codes: J: diseases of the respiratory system; R: symptoms, signs and abnormal clinical and laboratory findings not elsewhere classified.

Pneumonia: ICD-10 codes J12–J18 excluding J12.1. J (non-ARI): ICD-10 codes from group J excluding J00–J22 and J80. URI: ICD-10 codes J00–J06. Data on ICD-10 diagnosis codes come from the Norwegian Patient Registry and data on the main cause of admission (COVID-19 or other) from the Norwegian Pandemic Registry.

^a^ ICD-10 code U07.1 (COVID-19, virus identified).

^b^ Week 9/2020–6/2021: wild-type or ‘Wuhan’ variant dominant, COVID-19 vaccination programme started week 52/2020.

^c^ Week 7/2021–26/2021: Alpha variant dominant, second dose vaccination coverage reached 95% among persons ≥ 75 years, first dose coverage reached 66% among persons ≥ 18 years, few hospitalised COVID-19 patients vaccinated.

^d^ Week 27/2021–51/2021: Delta variant dominant, second dose vaccination coverage reached 89% among persons ≥ 18 years, increasing proportion of hospitalised COVID-19 patients vaccinated with at least two doses.

^e^ Week 52/2021–17/2022: Omicron variant dominant, third dose vaccination coverage reached 90% among persons ≥ 75 years and 66% among persons ≥ 18 years, majority of hospitalised COVID-19 patients vaccinated with three doses.

**Figure 4:**
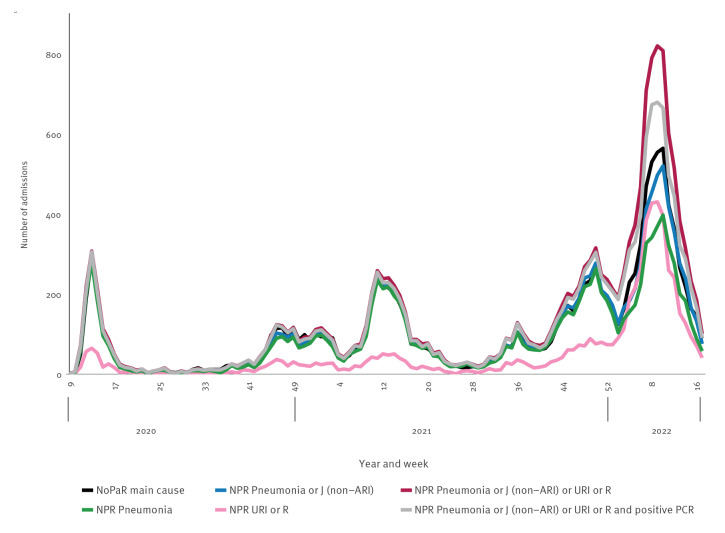
Weekly number of hospital admissions with confirmed COVID-19 as main cause or different international classification of diseases diagnosis code combinations among COVID-19 patients^a^, Norway, 17 February 2020–1 May 2022

ARI: acute respiratory infections; NoPaR: Norwegian Pandemic Registry; NPR: Norwegian Patient Registry; URI: upper respiratory infections.

Using data aggregated separately from each registry, the code combination pneumonia or J (non-ARI) (n = 12,221) most closely followed the trend in new admissions with COVID-19 as main cause in NoPaR (n = 12,638), although the same general trend was observed in all code combinations, aside from URI or R before the end of the study period (Figure 4).

International Classification of Diseases 10th revision (ICD-10) codes: J: diseases of the respiratory system; R: symptoms, signs and abnormal clinical and laboratory findings not elsewhere classified.

Pneumonia: ICD-10 codes J12–J18 excluding J12.1. J (non-ARI): ICD-10 codes from group J excluding J00–J22 and J80. URI: ICD-10 codes J00–J06.

^a^ ICD-10 code U07.1 (COVID-19, virus identified).

Positive PCR: positive test ≤ 14 days before admission until discharge. ICD-10 diagnosis codes come from NPR. Data on the main cause of admission (COVID-19 or other) come from NoPaR.

The data behind the figure are available in Supplement 2.

## Discussion

Despite different registration criteria in each registry, 90–100% of hospitalisation periods overlapped between U07.1 patients in NPR and patients in NoPaR, until late 2021 when the proportion of overlapping patients gradually decreased. Our results support previous studies in different settings that found high uptake and accurate use of U07.1 to identify COVID-19 patients earlier in the pandemic [28-31]. However, from late 2021, an increasing proportion of patients in NPR with a recent positive PCR test was not registered with U07.1, nor registered in NoPaR. Furthermore, over 1,600 U07.1 patients in NPR, predominantly admitted from late 2021, did not have a registered positive PCR test ≤ 60 days before admission until discharge. This suggests increasing registration of U07.1 for patients without a positive PCR test, contrary to national guidelines. Most U07.1 patients without a positive PCR test ≤ 60 days before admission until discharge did not have a recent rapid antigen test registered, however, self-tests are not registered in MSIS-labdatabase [22], thus these data must be interpreted with caution.

The decreasing overlap between the registries from late 2021 coincided with the Delta variant being superseded by the milder Omicron variant [5], increasing vaccination coverage [15] and the gradual scaling back of non-pharmaceutical interventions and testing in Norway. This consequently impacted the flow to, and management of, COVID-19-positive persons in hospital. Patients gradually became more spread out across hospitals, instead of being treated in specific wards usually under the care of infectious disease physicians. Our results suggest that this could have impacted the registration of new patients in NoPaR and ICD-10 codes in NPR, such that the two registries were identifying increasingly different patient cohorts and a decreasing proportion of all patients with a recent positive PCR test.

Since the start of the pandemic, Norway has disaggregated data on patients with COVID-19 as main cause of hospitalisation through a clinical assessment. The benefit of this disaggregation was clearly illustrated in late 2021, when the proportion of all COVID-19 patients hospitalised with COVID-19 as main cause fell markedly [15]. A similar trend was observed in other countries where ‘due to COVID-19’ was defined by diagnosis codes [9,11,32]. In our study, while certain ICD-10 code combinations closely followed the trend in new admissions with COVID-19 as main cause, the distribution of ICD-10 codes varied by age and time. The greater prominence of a wider range of codes among older age groups likely reflect a higher rate of underlying comorbidities. From late 2021 the frequency of pneumonia codes decreased in the age groups ≥ 18 years, potentially related to the increasing proportion of vaccinated patients [33-35]. In the same period, the proportion of patients 0–17 years admitted due to COVID-19 who were registered with a URI code increased, in line with findings from elsewhere during a period of increasing Omicron dominance [36] and the lifting of restrictions. After week 52/2021, the sensitivity of all code combinations including pneumonia was lower, compared with earlier periods. Clinical validation of the algorithm for admissions ‘due to COVID-19’ in Denmark also found that sensitivity decreased between Delta (95%) and early Omicron (87%) periods [9]. This highlights the challenge of using diagnosis codes for the surveillance of persons hospitalised due to COVID-19, not least the importance of age- and time-specific definitions. Other definitions of ‘due to COVID-19’ have also been proposed. For example, Public Health Scotland revised their definition to community-acquired hospital admissions with a positive PCR test from emergency admissions, as data on clinical discharge diagnoses were not timely enough for ongoing surveillance [14]. Surveillance of SARI, either EHR- or questionnaire-based, is an alternative standardised approach established in several European countries [25]. However, in the context of the surveillance of persons hospitalised due to COVID-19, one must consider how the definition of SARI may influence the sensitivity of the system. For example, in the EHR-based sentinel system in Germany, SARI is defined as patients admitted with ICD-10 codes J09–J22 [8]. This will miss patients admitted with an URI (J00–J06), which we observed in an increasing proportion of younger persons hospitalised due to COVID-19 since Omicron emerged.

Hospital admissions remain a central indicator for COVID-19 surveillance. Data collection should be sensitive and timely for public health action, representative, accurate, sustainable, collect relevant data on the patient cohort, integrated with, but able to distinguish between, different pathogens and not entail an unnecessary reporting burden. A diverse landscape of systems for this surveillance has emerged across Europe [2,8-13], with designs naturally tailored to the local setting, resource-availability and existing data collection infrastructure and practices. Looking forward, a European Union Joint Action ‘UNITED4Surveillance’ now aims to promote the integration, digitalisation and establishment of real-time surveillance systems in the European Union, including through EHR [37].

In light of this, our study provides clear examples of advantages and disadvantages with EHR-based systems for the surveillance of COVID-19 hospitalisations. Both NPR and NoPaR have national coverage, allow year-round surveillance, can be linked to other national registries to achieve integration between surveillance systems (e.g. molecular surveillance) and healthcare levels and may monitor additional severity outcomes like intensive care admission (not available for comparison in this study). These systems have thus outdated the sentinel, manual and weekly reporting Norway had for hospitalisation with another respiratory infection (influenza) before the pandemic. The pandemic registry collects COVID-19-specific data and data reporting has been timely [15]. However, in its current form, data registration in NoPaR entails an additional burden for hospitals and has more limited capacity to be integrated with the surveillance of other respiratory infections, as desired [25]. Surveillance systems based on electronic patient registries, like NPR, have the advantage of being part of the established data flow in hospitals and allow the integration of surveillance for different pathogens as well as syndromic and disease-specific components. Through linkage to laboratory results, they may also provide more complete data on persons hospitalised with COVID-19, as well as data on total and disease-specific hospital bed occupancy, which remains a recommended COVID-19 surveillance indicator [25]. However, the accuracy and timeliness of coding practices and changes in coding practices over time, must be considered. Also, NPR provides limited disease-specific data, such as on clinical severity and treatment. Furthermore, endeavours to accurately identify persons hospitalised due to COVID-19 will require ongoing validation to consider temporal changes in patient cohorts and virus characteristics. A future surveillance system would ideally encompass the benefits of both NoPaR and NPR, something Norwegian registries have demonstrated the feasibility of during the pandemic. A coordinated system will probably improve coverage by reducing the need for reporting via several systems.

Our study has several limitations. Firstly, different ICD-10 code combinations were registered among patients in both main cause categories. This highlights that clinicians may assess the main cause of admission differently for patients with similar diagnostic codes, leading to non-differential misclassification. This could potentially be alleviated by including more main cause categories beyond COVID-19/other [9]. Secondly, we cannot rule out that the decreasing overlap between U07.1 patients in NPR and patients in NoPaR from late 2021 affected our analysis of hospitalisation due to COVID-19 and the precision of our sensitivity and specificity estimates. Also, we did not have access to full ICD-10 codes for all diagnostic categories, which limited the exploration of whether more detailed code combinations could more precisely represent persons hospitalised due to COVID-19. We also only considered the distribution of ICD-10 codes in this analysis. However, other parameters could additionally inform more precise proxies. For example, in Denmark, the proportion of admission time related to certain diagnosis codes is considered [9]. Finally, we cannot rule out errors in data registration that may have influenced which patients and hospitalisation periods were able to be correctly linked. However, given the high degree of overlap and that most individuals have only been hospitalised with COVID-19 once, we do not believe this has unduly affected our results.

## Conclusion

In this study we compared data in two EHR in Norway on persons hospitalised with COVID-19 during the first 2 years of the pandemic. Our results show that the EHR provided an accurate picture of persons hospitalised with COVID-19, but also highlight challenges with using EHR data. This comparison has allowed more comprehensive understanding of the data in each EHR through different phases of the pandemic and can inform the ongoing development of surveillance systems for COVID-19 and in preparation for future pandemics.

## Supplementary Data

2049000 Supplementary Material 1

## Supplementary Data

90000 Supplementary Material 2
