# Supplementary material for: A comparison of two registry-based systems for the surveillance of persons hospitalised with COVID-19 in Norway, February 2020 to May 2022
Source: Euro Surveill. 2023 Aug 17;28(33):2200888. doi: 10.2807/1560-7917.ES.2023.28.33.2200888 (PMC10436689; doi:10.2807/1560-7917.ES.2023.28.33.2200888)
Supplement: Supplementary Material 1 [file 22-00888_WHITTAKER_Supplement1.pdf]

A comparison of two registry-based systems for the surveillance of persons hospitalised with COVID-19 in Norway, February 2020 to May 2022

Supplement 1

This supplementary material is hosted by Eurosurveillance as supporting information alongside the article "A comparison of two registry-based systems for the surveillance of patients hospitalised with COVID-19 in Norway, February 2020 to May 2022", on behalf of the authors, who remain responsible for the accuracy and appropriateness of the content. The same standards for ethics, copyright, attributions and permissions as for the article apply. Supplements are not edited by Eurosurveillance and the journal is not responsible for the maintenance of any links or email addresses provided therein.

1. ICD-10 diagnosis codes from the Norwegian patient registry available in this study

Table S1. ICD-10 diagnosis codes from the Norwegian patient registry available to the authors for the comparison of admissions with COVID-19 in the Norwegian patient registry and the Norwegian pandemic registry.

Disease group	ICD-10 code	Description
Certain infectious and parasitic diseases	A	
Whooping cough	A370	Whooping cough due to Bordetella pertussis
	A371	Whooping cough due to Bordetella parapertussis
	A378	Whooping cough due to other Bordetella species
	A379	Whooping cough, unspecified species
Certain infectious and parasitic diseases	B	
Neoplasms	C	
Neoplasms, Diseases of the blood and blood-forming organs and certain disorders involving the immune mechanism	D	
Endocrine, nutritional and metabolic diseases	E	
Mental, Behavioural and Neurodevelopmental disorders	F	
Diseases of the nervous system	G	
Diseases of the eye and adnexa, Diseases of the ear and mastoid process	H	
Otitis media	H650	Acute serous otitis media
	H651	Other acute nonsuppurative otitis media
	H659	Unspecified nonsuppurative otitis media
	H660	Acute suppurative otitis media
	H664	Suppurative otitis media, unspecified
	H669	Otitis media, unspecified
	H670	Otitis media in bacterial diseases classified elsewhere
	H671	Otitis media in viral diseases classified elsewhere
	H678	Otitis media in other diseases classified elsewhere
Diseases of the circulatory system	I	
Diseases of the respiratory system	J	

Acute upper respiratory infections	J00	Acute nasopharyngitis [common cold]
	J010	Acute maxillary sinusitis
	J011	Acute frontal sinusitis
	J012	Acute ethmoidal sinusitis
	J013	Acute sphenoidal sinusitis
	J014	Acute pansinusitis
	J018	Other acute sinusitis
	J019	Acute sinusitis, unspecified
	J020	Streptococcal pharyngitis
	J028	Acute pharyngitis due to other specified organisms
	J029	Acute pharyngitis, unspecified
	J030	Streptococcal tonsillitis
	J038	Acute tonsillitis due to other specified organisms
	J039	Acute tonsillitis, unspecified
	J040	Acute laryngitis
	J041	Acute tracheitis
	J042	Acute laryngotracheitis
	J050	Acute obstructive laryngitis [croup]
	J051	Acute epiglottitis
	J060	Acute laryngopharyngitis
	J068	Other acute upper respiratory infections of multiple sites
	J069	Acute upper respiratory infection, unspecified
Influenza	J09	Influenza due to identified zoonotic or pandemic influenza viruses
	J100	Influenza due to identified seasonal influenza virus with pneumonia
	J101	Influenza due to identified seasonal influenza virus with other respiratory manifestations
	J108	Influenza due to identified seasonal influenza virus with other manifestations
	J110	Influenza due to unidentified influenza virus with pneumonia
	J111	Influenza due to unidentified influenza virus with other respiratory manifestations
	J118	Influenza due to unidentified influenza virus with other manifestations
Pneumonia	J120	Adenoviral pneumonia
	J121	Respiratory syncytial virus pneumonia
	J122	Parainfluenza virus pneumonia
	J123	Human metapneumovirus pneumonia
	J128	Other viral pneumonia
	J129	Viral pneumonia, unspecified
	J13	Pneumonia due to Streptococcus pneumoniae
	J14	Pneumonia due to Hemophilus influenzae
	J150	Pneumonia due to Klebsiella pneumoniae
	J151	Pneumonia due to Pseudomonas
	J152	Pneumonia due to staphylococcus
	J153	Pneumonia due to streptococcus, group B
	J154	Pneumonia due to other streptococci
	J155	Pneumonia due to Escherichia coli
	J156	Pneumonia due to other Gram-negative bacteria
	J157	Pneumonia due to Mycoplasma pneumoniae
	J158	Pneumonia due to other specified bacteria
	J159	Unspecified bacterial pneumonia

	J160	Chlamydial pneumonia
	J168	Pneumonia due to other specified infectious organisms
	J170	Pneumonia in bacterial diseases classified elsewhere
	J171	Pneumonia in viral diseases classified elsewhere
	J172	Pneumonia in fungal diseases
	J173	Pneumonia in parasitic diseases
	J178	Pneumonia in other diseases classified elsewhere
	J180	Bronchopneumonia, unspecified organism
	J181	Lobar pneumonia, unspecified organism
	J182	Hypostatic pneumonia, unspecified organism
	J188	Other pneumonia, unspecified organism
	J189	Pneumonia, unspecified organism
Other acute lower respiratory infections	J200	Acute bronchitis due to Mycoplasma pneumoniae
	J201	Acute bronchitis due to Hemophilus influenzae
	J202	Acute bronchitis due to streptococcus
	J203	Acute bronchitis due to coxsackievirus
	J204	Acute bronchitis due to parainfluenza virus
	J205	Acute bronchitis due to respiratory syncytial virus
	J206	Acute bronchitis due to rhinovirus
	J207	Acute bronchitis due to echovirus
	J208	Acute bronchitis due to other specified organisms
	J209	Acute bronchitis, unspecified
	J210	Acute bronchiolitis due to respiratory syncytial virus
	J211	Acute bronchiolitis due to human metapneumovirus
	J218	Acute bronchiolitis due to other specified organisms
	J219	Acute bronchiolitis, unspecified
	J22	Unspecified acute lower respiratory infection
	J80	Acute respiratory distress syndrome in adults (ARDS)
Diseases of the digestive system	K	
Diseases of the skin and subcutaneous tissue	L	
Diseases of the musculoskeletal system and connective tissue	M	
Diseases of the genitourinary system	N	
Pregnancy, childbirth and the puerperium	O	
Certain conditions originating in the perinatal period	P	
Congenital malformations, deformations and chromosomal abnormalities	Q	
Symptoms, signs and abnormal clinical and laboratory findings, not elsewhere classified	R	
Injury, poisoning and certain other consequences of external causes	S	
Injury, poisoning and certain other consequences of external causes	T	
Codes for special purposes	U	
COVID-19	U071	COVID-19, virus identified
	U072	COVID-19, virus not identified
External causes of morbidity	V	
External causes of morbidity	W	
External causes of morbidity	X	
External causes of morbidity	Y	
Factors influencing health status and contact with health services	Z	

2. Number of COVID-19 patients in each registry by time, age and sex

Table S2. Number of admissions and characteristics of patients with confirmed COVID-19 in the Norwegian Pandemic Registry and Norwegian Patient Registry, by period, Norway, 17 February 2020–1 May 2022.

Period (week/year)	Registry	Number of admissions	Median length of stay in days (IQR)	Number of patients	Median age in years (IQR)	Male (%)
**9/2020 – 6/2021**	NoPaR	3,425	5 (2–9)	3,242	60 (47–74)	1,851 (57%)
	NPR (U07.1)	3,379	5 (2–10)	3,191	61 (47–75)	1,832 (57%)
**7/2021 – 26/2021**	NoPaR	2,501	4 (2–9)	2,392	52 (42–64)	1,439 (60%)
	NPR (U07.1)	2,553	4 (2–9)	2,408	53 (42–65)	1,447 (60%)
**27/2021 – 51/2021**	NoPaR	3,553	5 (2–10)	3,410	60 (42–76)	1,879 (55%)
	NPR (U07.1)	3,667	5 (2–10)	3,507	59 (41–76)	1,926 (55%)
**52/2021 – 17/2022**	NoPaR	9,771	3 (1–5)	9,453	67 (41–79)	5,086 (54%)
	NPR (U07.1)	11,216	3 (1–6)	10,817	67 (40–80)	5,710 (53%)

NoPaR: Norwegian Pandemic Registry; NPR: Norwegian Patient Registry; IQR: Interquartile range

3. **Prevalence of ICD-10 diagnosis codes and code combinations for admissions in NoPaR who were not registered with U07.1 in NPR**

For admissions with main cause COVID-19 and other, U07.1 was the most common ICD-10 code registered (11,380/11,803 (96%) and 5,143/6,206 (83%), respectively). Figure S1 presents the distribution of ICD-10 not registered with U07.1 in NPR among the 423 admissions with main cause COVID-19 and 1063 with another main cause of admission in NoPaR.

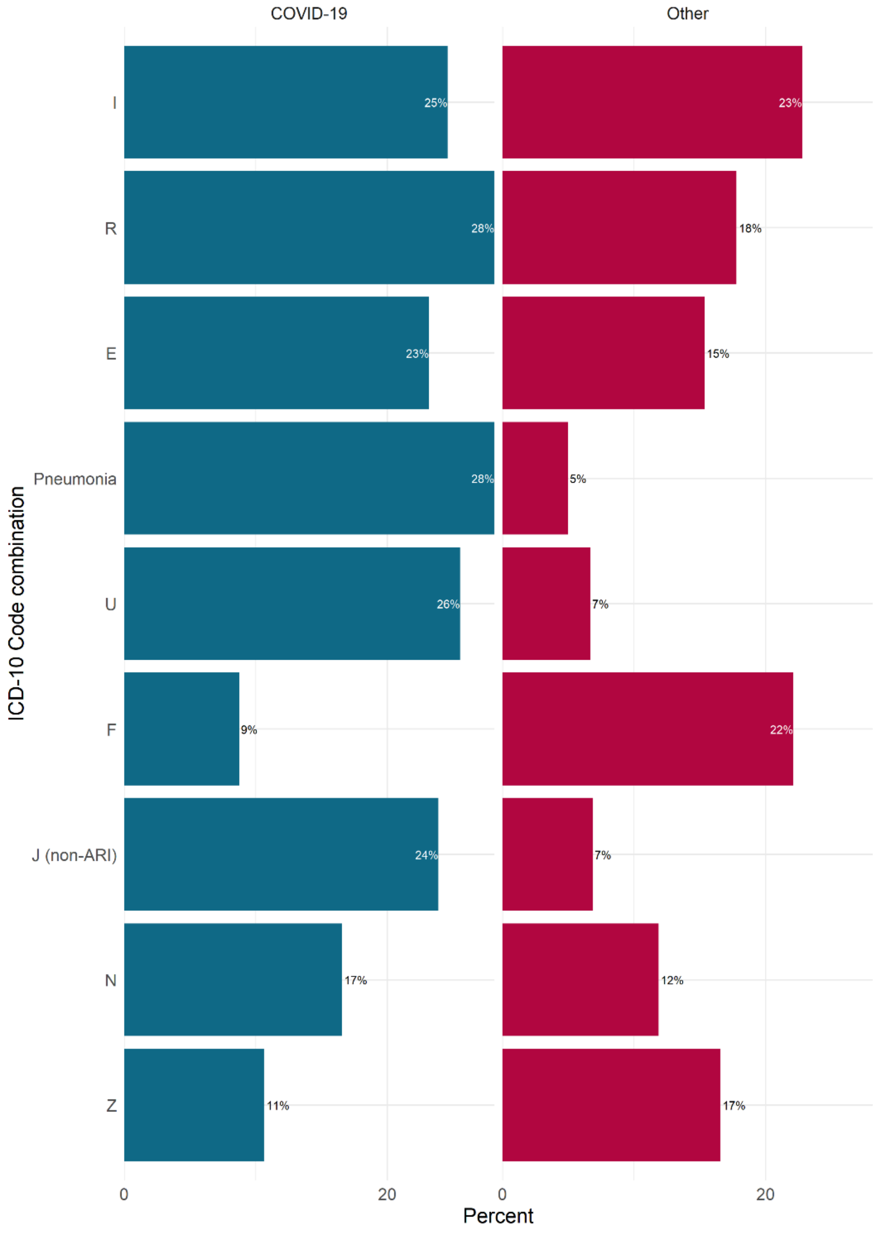

Figure S1. Prevalence of ICD-10 diagnosis codes and code combinations by clinically assessed main cause of admission (COVID-19 or other), Norway, excluding patients registered with U07.1, 17 February 2020–1 May 2022.

Pneumonia: ICD-10 codes J12-J18, excluding J12.1. J (non-ARI): respiratory diseases other than acute respiratory infections; ICD-10 codes from group J excluding J00-J22 and J80. For descriptions of other codes, see table S1. ICD-10 diagnosis codes come from the Norwegian patient registry. Data on the main cause of admission (COVID-19 or other) come from the Norwegian pandemic registry. Note that code combinations may overlap. Only codes and code combinations with at least 15% of patients for either main cause are presented.

4. Prevalence of ICD-10 diagnosis codes and code combinations by main cause of admission, age group and period

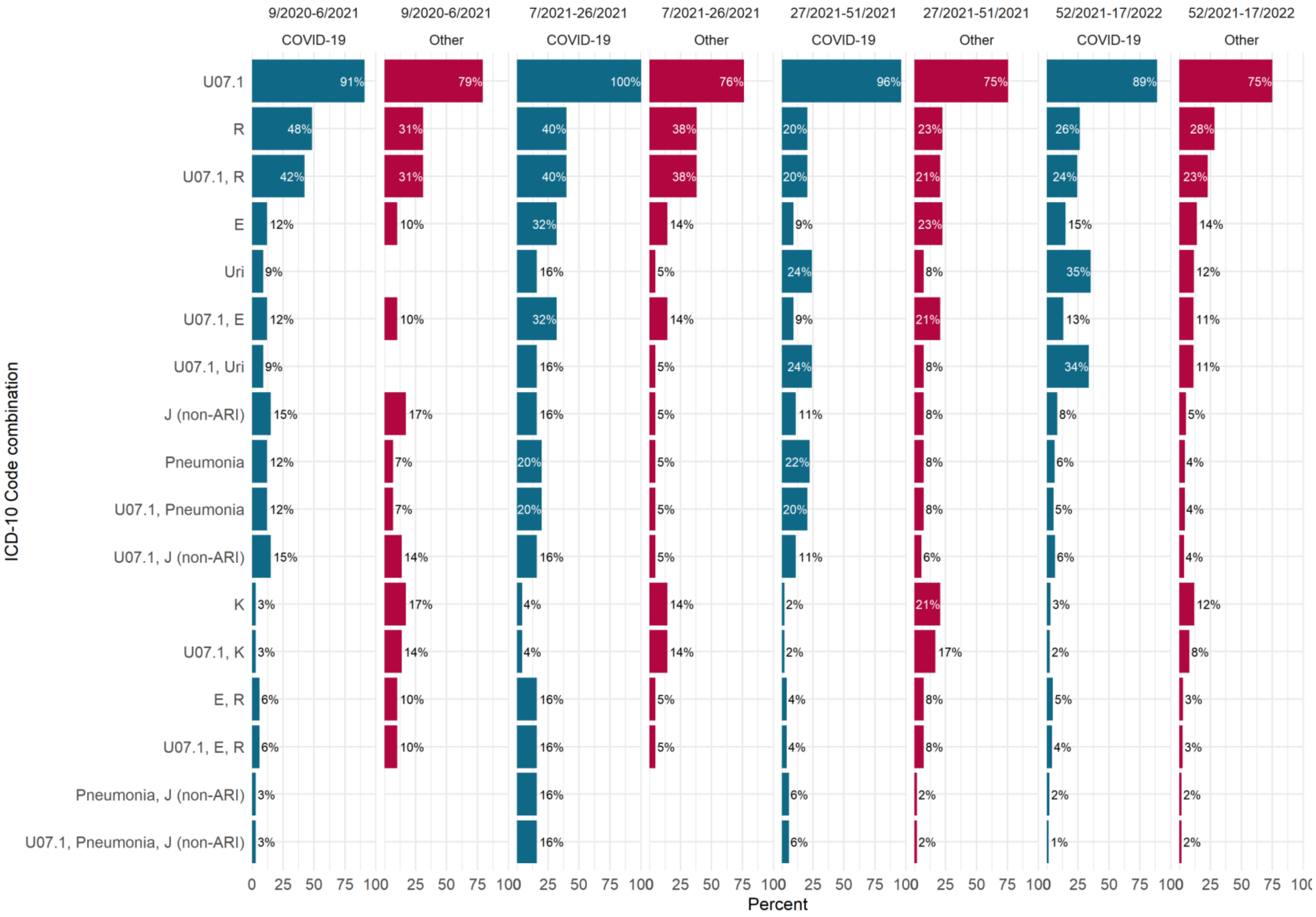

Figure S2. Prevalence of ICD-10 diagnosis codes and code combinations by clinically assessed main cause of admission (COVID-19 or other) and period (week/year), patients aged 0–17 years, Norway, 17 February 2020–1 May 2022.

Pneumonia: ICD-10 codes J12-J18, excluding J12.1. J (non-ARI): respiratory diseases other than acute respiratory infections; ICD-10 codes from group J excluding J00-J22 and J80. Uri: upper respiratory infections; ICD-10 codes J00-J06. For descriptions of other codes, see table S1. ICD-10 diagnosis codes come from the Norwegian patient registry. Data on the main cause of admission (COVID-19 or other) come from the Norwegian pandemic registry. Note that code combinations may overlap. Only codes and code combinations with at least 15% of patients for either main cause in any period are presented.

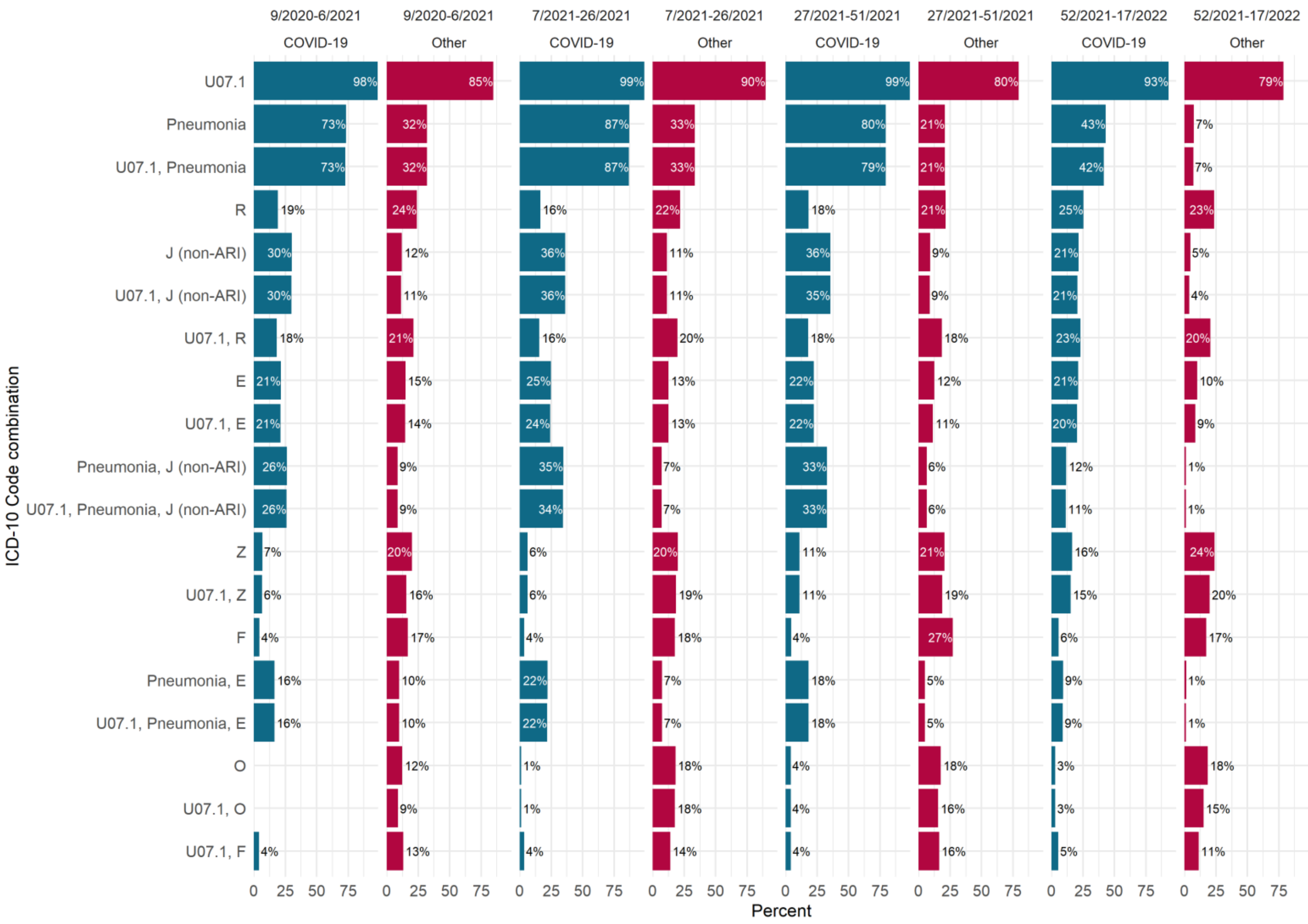

Figure S3. Prevalence of ICD-10 diagnosis codes and code combinations by clinically assessed main cause of admission (COVID-19 or other) and period (week/year), patients aged 18–54 years, Norway, 17 February 2020–1 May 2022.

Pneumonia: ICD-10 codes J12-J18, excluding J12.1. J (non-ARI): respiratory diseases other than acute respiratory infections; ICD-10 codes from group J excluding J00-J22 and J80. Uri: upper respiratory infections; ICD-10 codes J00-J06. For descriptions of other codes, see table S1. ICD-10 diagnosis codes come from the Norwegian patient registry. Data on the main cause of admission (COVID-19 or other) come from the Norwegian pandemic registry. Note that code combinations may overlap. Only codes and code combinations with at least 15% of patients for either main cause in any period are presented.

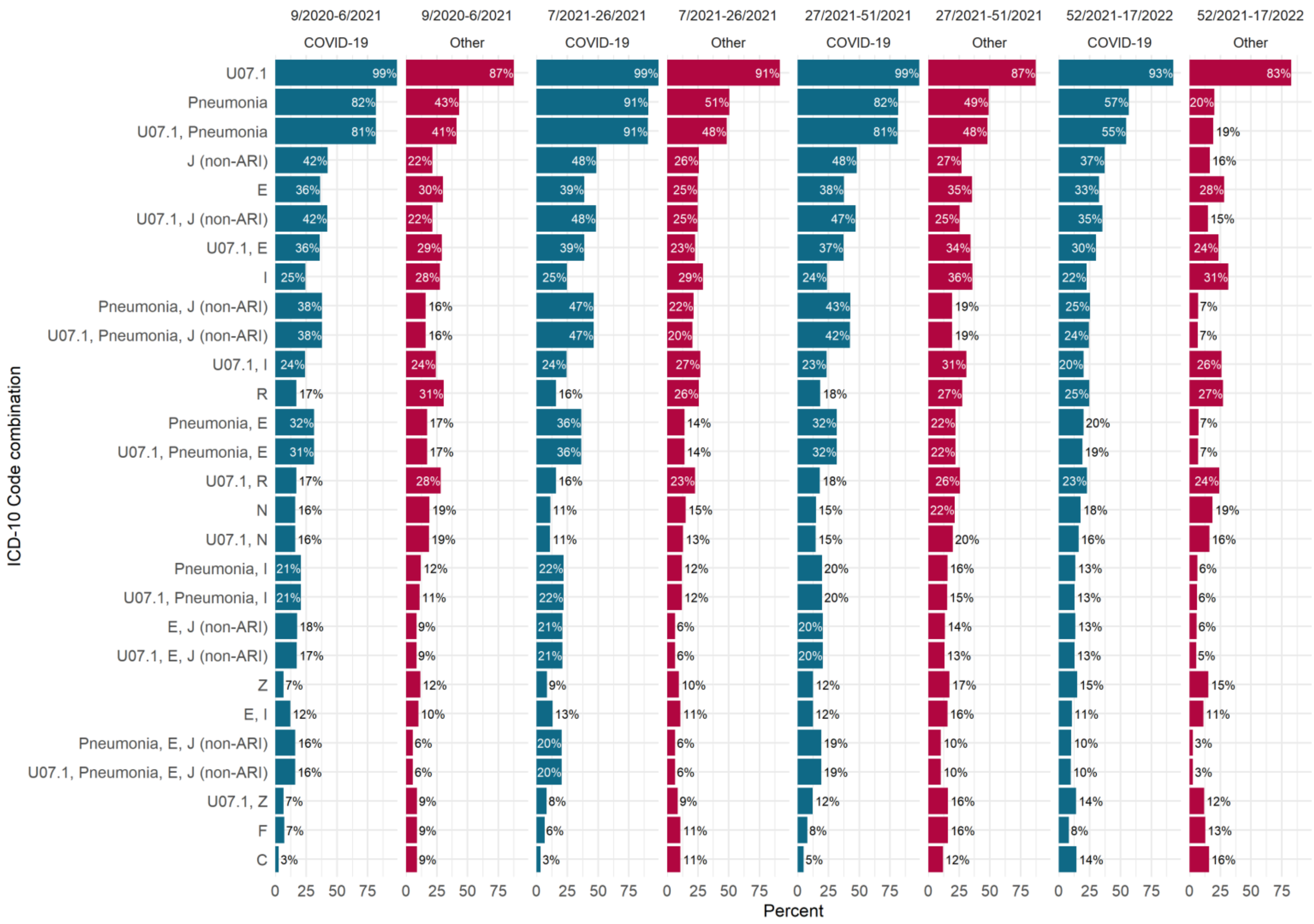

Figure S4. Prevalence of ICD-10 diagnosis codes and code combinations by clinically assessed main cause of admission (COVID-19 or other) and period (week/year), patients aged 55–74 years, Norway, 17 February 2020–1 May 2022.

Pneumonia: ICD-10 codes J12-J18, excluding J12.1. J (non-ARI): respiratory diseases other than acute respiratory infections; ICD-10 codes from group J excluding J00-J22 and J80. Uri: upper respiratory infections; ICD-10 codes J00-J06. For descriptions of other codes, see table S1. ICD-10 diagnosis codes come from the Norwegian patient registry. Data on the main cause of admission (COVID-19 or other) come from the Norwegian pandemic registry. Note that code combinations may overlap. Only codes and code combinations with at least 15% of patients for either main cause in any period are presented.

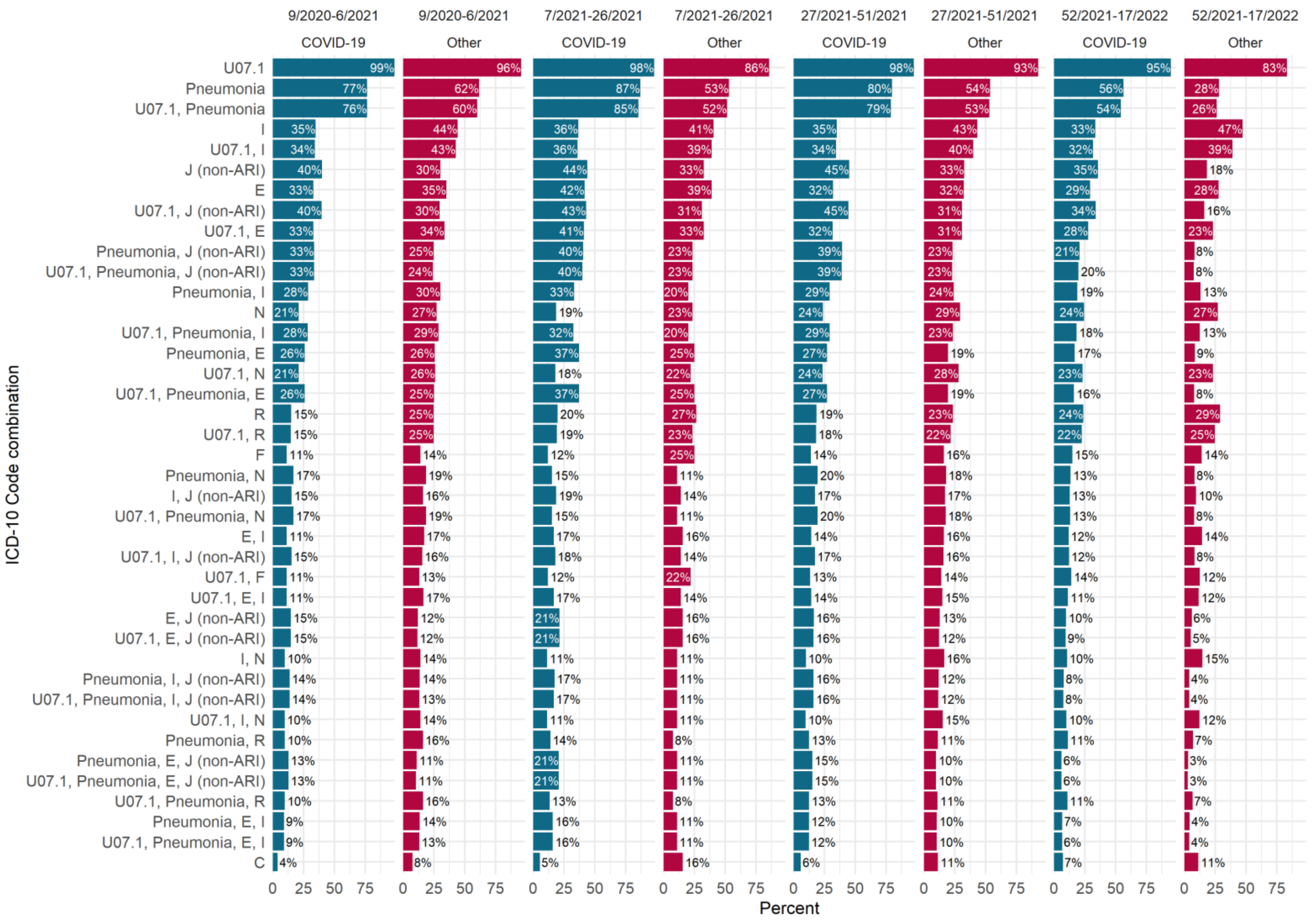

Figure S5. Prevalence of ICD-10 diagnosis codes and code combinations by clinically assessed main cause of admission (COVID-19 or other) and period (week/year), patients aged ≥75 years, Norway, 17 February 2020–1 May 2022.

Pneumonia: ICD-10 codes J12-J18, excluding J12.1. J (non-ARI): respiratory diseases other than acute respiratory infections; ICD-10 codes from group J excluding J00-J22 and J80. Uri: upper respiratory infections; ICD-10 codes J00-J06. For descriptions of other codes, see table S1. ICD-10 diagnosis codes come from the Norwegian patient registry. Data on the main cause of admission (COVID-19 or other) come from the Norwegian pandemic registry. Note that code combinations may overlap. Only codes and code combinations with at least 15% of patients for either main cause in any period are presented.
